# The Role of Measurement in Identifying High-Intensity Secure Message Senders Among Older Adults: Observational Study

**DOI:** 10.2196/93641

**Published:** 2026-10-21

**Authors:** Aleksandra Wec, Jenni Seale Reiff, Kelly T Gleason, Chanee D Fabius, John McGready, Jennifer L Wolff

**Affiliations:** 1 Division of General Internal Medicine, Icahn School of Medicine at Mount Sinai, One Gustave L. Levy Place, Box 1087, New York, NY, 10029, United States, 1 908-899-3882; 2 Division of Geriatrics and Gerontology, Department of Medicine, Emory University School of Medicine, Atlanta, GA, United States; 3 VA Atlanta Healthcare System, Atlanta, GA, United States; 4 Johns Hopkins School of Nursing, Johns Hopkins University, Baltimore, MD, United States; 5 Department of Health Policy and Management, Johns Hopkins Bloomberg School of Public Health, Johns Hopkins University, Baltimore, MD, United States; 6 Department of Biostatistics, Johns Hopkins Bloomberg School of Public Health, Johns Hopkins University, Baltimore, MD, United States

**Keywords:** patient portals, electronic health records, secure messaging, health services use, health care provider–patient interactions, older adults

## Abstract

**Background:**

Use of secure messaging within the patient portal has grown rapidly over recent years, yet clinical workflows, staffing models, and billing practices have generally not been comprehensively adapted to support this increasing volume of messages. Little is known about the characteristics of patients who are high-volume message senders, and we lack a nuanced understanding of patient messaging intensity beyond measures accounting for sheer volume, limiting health system efforts to anticipate message-related workflow demands and allocate resources effectively.

**Objective:**

Our objective was to characterize older adult patients (aged ≥65 years) with high secure messaging volume, examining both patient characteristics and other aspects of their messaging intensity, such as messaging frequency, length, and messaging use relative to patient portal logins and health care encounters.

**Methods:**

We analyzed electronic medical record and patient portal data from a large academic health system, encompassing 16,023 older adults who sent 199,981 messages over a 12-month period. We developed 5 measures to account for secure messaging intensity. Our primary measure of messaging intensity was based on message volume; high-volume message senders were identified using outlier analysis based on patients’ aggregate number of messages sent during the observation period. Additional measures of messaging intensity included identifying individuals with concentrated periods of messaging, message length (character count), a ratio of messages to portal logins, and a ratio of messages to health care encounters. We compared sociodemographic characteristics, health status, and messaging intensity of high-volume secure messaging senders with those of other message senders.

**Results:**

Of 16,023 older adult patients who sent at least 1 message during the observation period, 1298 (8.1%) were classified as high-volume message senders; these patients accounted for 39.7% (79,475/199,981) of total messages. High-volume message senders, compared with all other message senders, were more likely to be White (1044/1298, 80.4% vs 10,670/14,725, 72.5%; *P*<.001), have higher comorbidity scores (2.6 vs 1.8; *P*<.001), and higher incidence of cancer (465/1298, 35.8% vs 3354/14,725, 22.8%; *P*<.001) and dementia (107/1298, 8.2% vs 889/14,725, 6.1%; *P*<.002). High-volume message senders were also more likely to be identified as having concentrated periods of messaging, to send longer messages, and to send more messages in relation to patient portal logins and health care encounters.

**Conclusions:**

High-volume message senders represent a small but distinct group of older patients who send a large share of messages to clinicians. Using multiple measures of messaging intensity can provide additional context about patient messaging behavior and help health systems redesign workflows and care delivery models to meet patient communication needs while facilitating sustainable workloads for clinicians.

## Introduction

Digital health technologies have transformed health care delivery by facilitating transparency and enabling patients to have a greater sense of ownership over their health information [1-5]. Through the patient portal, patients can view their health records, test results, and visit notes, and communicate asynchronously with clinicians via secure messaging [6,7]. Secure messaging is one of the most frequently used features of the patient portal [8], with substantial year-over-year increases in message volume [9-13]. Evidence shows that secure messaging is time-saving and convenient for patients [5,14] and may facilitate care coordination [15,16] and self-management of chronic conditions [17-19]. While secure messaging use continues to grow [13], clinical workflows [20], staffing models [20,21], and billing policies have generally not evolved at the same pace [22-24]. The result for clinicians is significant; high message volumes drive workflow inefficiencies [2,25], task switching and multitasking [26], and burnout, with clinicians often managing inboxes outside of regular work hours [27-31]. Billing codes for responses to patients’ messages are available, but their adoption has been limited [22,32]. Health systems are working on implementing and evaluating workflow innovations, including new staffing models and AI-assisted message management, although many approaches remain in early stages [8,20,21,33-35]. A better understanding of messaging volume and intensity may help health systems anticipate patients’ communication needs, optimize clinical workflows, and allocate resources to support both high-quality patient-clinician communication and sustainable clinician workloads.

Previous analyses of patient-initiated secure messaging have typically examined the demographic and health characteristics of message senders as a single group, without distinguishing between those who send many messages and those who send only one [36-39]. The literature that has examined volume has generally done so narrowly, using continuous measures [40] or categorizing senders into “high” vs “low” groups [41,42]. A more data-driven and nuanced examination of messaging patterns and behaviors could inform targeted strategies to more effectively manage messaging volume and ensure patients are being well supported.

To address this gap, we conducted an analysis to comprehensively examine secure messaging intensity among older adults at an academic health system. Our aim was to identify high-volume message senders, profile their characteristics, and further describe their messaging intensity based on their message frequency, message length, and the number of messages sent relative to patient portal logins and health care encounters.

## Methods

### Sample Overview

We drew on a sample of older adult patients aged 65 years and older who received care at a large academic health system between October 1, 2021, and September 30, 2022, as described in previous work [43,44]. Our sample comprised 16,023 unique patients, all of whom sent at least 1 secure “medical advice request” message during the year via a patient portal (MyChart). A medical advice request is one of several options used to categorize patient messages in this patient portal; this message type was selected because it is the most open-ended type of patient message and most likely to be directed to clinicians. Other patient message types (eg, “appointment scheduling request”) are likely to be routed to other staff (such as front desk staff) and were not included in our sample.

### Measuring Patient Sociodemographic Characteristics and Health Status

Patient demographic characteristics drawn from the electronic medical record (EMR) included patient age in years, sex (male or female), race (White, Black, Asian or Pacific Islander, or other: any other race), ethnicity (Hispanic or non-Hispanic), marital status (married or not married, divorced or legally separated, single, or widowed), and whether the patient identified English as their preferred language.

Because our EMR data did not include measures that reflect socioeconomic status, we used patient addresses and 9-digit zip codes to calculate an Area Deprivation Index (ADI) score for each patient, as a proxy measure of socioeconomic disadvantage. An ADI score is composed of 17 US Census–based measures that collectively characterize the socioeconomic disadvantage of a neighborhood [45,46]. ADI scores range from 1 to 100, with higher scores indicating a higher level of disadvantage in that neighborhood.

We calculated the Charlson Comorbidity Index (CCI) for each patient from *International Statistical Classification of Diseases, Tenth Revision* (*ICD-10*) codes to measure overall disease burden, with higher scores indicating greater comorbidity [45,47]. Finally, we identified the presence of a diagnosis of 3 specific health conditions: cancer, dementia, and diabetes. We focused on diabetes because of the robust literature describing patient portal and secure messaging use for this condition [17,48-50]. Dementia and cancer were included because of the complexity of care involved in these conditions and the relevance of care coordination, which secure messaging can facilitate [15].

### Measuring Messaging Intensity

We constructed 5 measures reflecting secure messaging intensity from time-stamped patient portal and EMR data. We conducted outlier analysis using the IQR method [51] to identify “high-intensity” message senders according to each measure. For each measure, the upper threshold was determined as quartile 3+1.5×IQR. Any values equaling or exceeding this upper bound were classified as upper outliers and thus “high-intensity” (Multimedia Appendix 1). Detailed descriptions and rationale for each measure are included in Multimedia Appendix 2 [10,52-54]. We briefly describe each of the 5 measures here.

First, the volume measure identifies high-intensity message senders based on the aggregate number of messages sent during the observation period. Any patient identified as an outlier by the IQR method is referred to as a “high-volume” message sender. This serves as our primary measure of high-intensity message sending and defines our main group of interest for analysis.

Second, the messaging episodes measure captures “episodes” of concentrated messaging. To conservatively allow time for responses, messages sent less than 10 days apart were considered part of the same messaging episode. Within each episode, we count the number of messages and identify “high-intensity episodes” using the IQR method.

Third, the length (character count) measure calculates the number of characters per message to identify messages that had a disproportionately high number of characters using the IQR method.

Fourth, the ratio of messaging to portal logins (message-to-portal ratio) measure is operationalized as the ratio of the number of secure messages to the number of patient portal logins during the observation period and reflects disproportionate messaging relative to portal use.

Fifth, the ratio of messages to encounters (message-to-encounter ratio) measure reflects a ratio of the number of secure messages to the number of in-person encounters during the year.

For each measure, we categorized patients as “high-intensity” or “other.” Beyond identifying high-intensity senders for the messaging episode measure (those who had at least 1 episode with a “high” number of messages) and the length measure (those who sent at least 1 disproportionately “long” message), we also report the number of messaging episodes meeting the criteria for “high” messaging volume by patient and the number of messages meeting the criteria for being “long,” by patient. The measures expressed as ratios are reported as continuous measures. We also separately report the number of portal logins and encounters.

### Analytic Approach

#### Characteristics of High-Volume Message Senders

We first identified patients as high-intensity message senders based on aggregate volume. We then described the messaging behavior of high-volume senders using the other 4 measures. We conducted tests to identify statistically significant differences between high-volume senders and all other message senders with respect to patient characteristics and message-sending behavior based on the messaging episodes, length, and ratios of patient portal use and encounters to messages. Tests of statistical significance included chi-square tests for categorical variables and 2-tailed *t* tests for continuous variables. We also fitted a multivariable logistic regression model to examine patient characteristics associated with high-volume messaging.

#### Correlation and Convergence of Measures

To evaluate the discriminative validity of using these 5 measures of messaging intensity to capture unique elements of messaging behavior, we examined correlations using Pearson correlation coefficients. We followed the standard interpretation of coefficients between 0.70 and 1 as indicating strong correlation, coefficients between 0.40 and 0.69 as indicating moderate correlation, and coefficients below 0.40 as indicating weak or no correlation [55].

### Ethical Considerations

This study was approved by the Johns Hopkins School of Medicine Institutional Review Board (IRB00303190). All data were housed and analyzed within a secure, access-controlled computing platform provided by Johns Hopkins University.

## Results

### Sample Characteristics

The sample consisted of 16,023 unique patients who sent a total of 199,981 messages over the 12-month observation period (Table 1). The average number of messages sent per patient was 12.5 (SD 20.4), with a median of 6 (IQR 12) messages (Multimedia Appendix 1). Patients had an average age of 73.4 (SD 7.0) years and were predominantly White (n=11,714, 73.1%), non-Hispanic (n=15,585, 95.4%), and married (n=10,528, 65.7%), and predominantly had a preferred language of English (n=15,719, 98.1%; Table 1). The average ADI among patients was 65.3 (SD 20.1). The average comorbidity score (CCI) was 1.9 (SD 1.7). In total, 26.1% (n=4174) of patients in our sample had diagnosed diabetes, 23.9% (n=3819) had diagnosed cancer, and 6.2% (n=996) had diagnosed dementia.

**Table 1.** Patient characteristics, high-volume versus all other senders^a^.

	Full sample (n=16,023)	High-volume message senders (n=1298)	All other senders (n=14,725)	*P* value^b^
Age (y), mean (SD)	73.4 (7.0)	73.9 (6.9)	73.4 (7.1)	.006
Sex, n (%)				.15
Male	7197 (44.9)	558 (43)	6639 (45.1)	
Female	8826 (55.1)	740 (57)	8086 (54.9)	
Race, n (%)				<.001
White	11,714 (73.1)	1044 (80.4)	10,670 (72.5)	
Black	2793 (17.4)	149 (11.5)	2644 (18)	
Asian or Pacific Islander	403 (2.52)	11 (0.9)	392 (2.7)	
Other or unknown	1113 (7)	94 (7.2)	1019 (6.9)	
Ethnicity, n (%)				
Non-Hispanic	15,285 (95.4)	1260 (97.1)	14,025 (95.3)	.007
Marital status, n (%)				.23
Married	10,528 (65.7)	825 (63.6)	9703 (65.9)	
Not married^c^	45495(34.3)	473 (36.4)	5022(341)	
Area Deprivation Index^d^, mean (SD)	65.3 (20.1)	64.4 (21.6)	65.4 (20.0)	.07
Comorbidity score^e^, mean (SD)	1.9 (1.7)	2.6 (1.9)	1.8 (1.7)	<.001
Health care conditions, n (%)				
Diabetes	4174 (26.1)	365 (28.1)	3809 (25.9)	.08
Cancer	3819 (23.9)	465 (35.8)	3354 (22.8)	<.001
Dementia	996 (6.2)	107 (8.2)	889 (6.1)	<.002

a A total of 199,981 patient-generated messages were included, of which 79,475 were sent by high-volume message senders and 120,506 were sent by all other message senders. Most messages (72.9%) were sent during business hours (9 AM to 5 PM). The 199,981 patient-generated messages corresponded to 140,380 clinician-generated messages (clinician messages are not included in this analysis). The higher number of patient messages partly reflects brief patient “thank you” messages and does not necessarily indicate unaddressed messages.

b Tests of statistical significance included *t* tests for continuous variables and chi-square tests for categorical variables.

c Not married consisted of widowed, divorced, legally separated, or single.

d Area Deprivation Index scores range from 1 to 100, with higher scores indicating a higher level of disadvantage.

e Comorbidity score was calculated using the Charlson Comorbidity Index (CCI).

### High-Volume Message Senders Versus All Others

High-volume message senders were defined as patients who sent 33 or more messages during the 12-month observation period (Multimedia Appendix 1). High-volume message senders were more likely to be White (1044/1298, 80.4% vs 10,670/14,725, 72.5%) and older than other message senders (mean age 73.9, SD 6.9 years vs 73.4, SD 7.1 years; *P*=.006) but were otherwise similar with respect to sex, marital status, and ADI. High-volume message senders had a greater mean comorbidity score (2.6, SD 1.9 vs 1.8, SD 1.7; *P*<.001) and were more likely to have been diagnosed with cancer (465/1298, 35.8% vs 3354/14,725, 22.8%; *P*<.001) and dementia (107/1298, 8.3% vs 889/14,725, 6.1%; *P*<.002) than other message senders.

In adjusted analyses, older age (adjusted odds ratio [aOR] 0.99, 95% CI 0.98‐1.00), female sex (aOR 0.85, 95% CI 0.75‐0.96), and Black (aOR 0.45, 95% CI 0.37‐0.53) or Asian (aOR 0.29, 95% CI 0.16‐0.53) race (compared with White) and having a diagnosis of diabetes (aOR 0.63, 95% CI 0.54‐0.74) were associated with lower odds of being a high-volume message sender (Table 2). Higher comorbidity burden (aOR 1.33, 95% CI 1.28‐1.38) and a diagnosis of cancer (aOR 1.30, 95% CI 1.14‐1.48) were associated with greater odds of high-volume messaging. Ethnicity, marital status, dementia diagnosis, and ADI were not associated with high-volume messaging.

**Table 2.** Adjusted odds of being a high-volume message sender.

	Adjusted odds ratio (95% CI)	*P* value
Age (y)	0.99 (0.98‐1.00)	.05
Female (reference: male)	0.85 (0.75‐0.96)	.01
Race (reference: White)		
Black	0.45 (0.37‐0.53)	<.001
Asian or Pacific Islander	0.29 (0.16‐0.53)	<.001
Other or unknown	0.98 (0.71‐1.36)	.91
Non-Hispanic (reference: Hispanic)	0.87 (0.73‐1.06)	.16
Not married^a^ (reference: married)	0.98 (0.90‐1.08)	.70
Area Deprivation Index^b^	0.99 (0.99‐1.01)	.12
Comorbidity score^c^	1.33 (1.28‐1.38)	<.001
Health care conditions		
Diabetes	0.63 (0.54‐0.74)	<.001
Cancer	1.30 (1.14‐1.48)	<.001
Dementia	1.07 (0.86‐1.34)	.55

a Not married consisted of widowed, divorced, legally separated, or single.

b Area Deprivation Index scores range from 0 to 100, with higher scores indicating a higher level of disadvantage.

c Comorbidity score was calculated using the Charlson Comorbidity Index (CCI).

### Messaging Behaviors

High-volume message senders comprised a small proportion of total message senders (1298/16,023, 8.1%; Figure 1) but sent 39.7% (79,475/199,981) of all messages. High-volume message senders on average sent 61.2 (SD 43.6) messages per year, nearly 8 times more than other senders, who averaged 8.2 (SD 7.5) messages annually (Table 3).

Nearly 9 in 10 (1160/1298, 89.4%) high-volume message senders experienced at least 1 high-intensity messaging episode (8.5 or more messages per episode), as compared with just 6% (883/14,725) of other senders (*P*<.001; Table 3). The number of high-intensity messaging episodes was significantly greater among high-volume message senders (mean of 2.4, SD 1.9 vs 0.1, SD 0.3; *P*<.001).

This analysis identified a total of 11,186 long messages (messages equal to or over 787.5 characters), representing approximately 5.6% of the total messages. High-volume senders were responsible for more than half of long messages (Figure 1). About 68% (872/1298) of high-volume message senders authored at least 1 long message, compared with 19.6% (2,883/14,725) of other senders (*P*<.001; Table 3). On average, high-volume message senders authored 3.8 (SD 13.0) long messages; in contrast, other senders on average sent less than 1 long message (0.3, SD 1.0).

**Figure 1.**
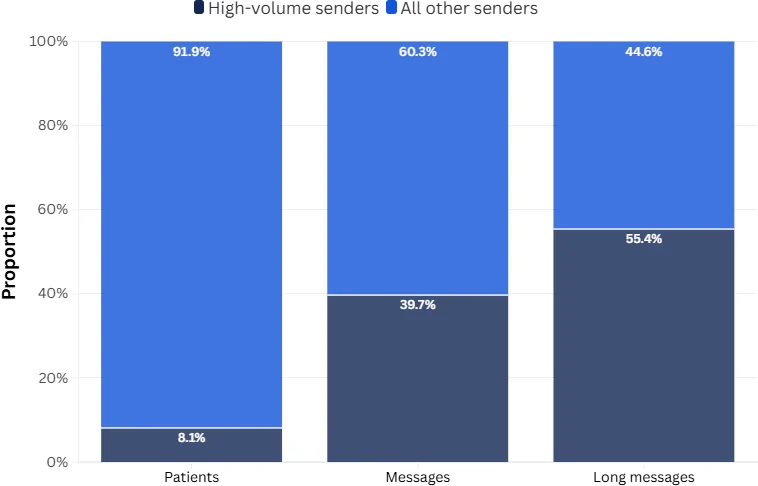
Proportion of total messages and long messages sent by high-volume message senders versus all other message senders. High-volume senders were identified based on the aggregate number of messages sent during the observation period; those who sent 33 or more messages were considered high-volume based on outlier analysis. Long messages were those with the number of characters equaling or exceeding the upper outlier threshold calculated (≥787.5 characters).

High-volume secure message senders exhibited significantly greater overall patient portal use (Table 3). They had a higher message-to-portal login ratio (0.52 vs 0.15; *P*<.001) and sent more messages relative to health care encounters (message-to-encounter ratio 3.3 vs 1.3; *P*<.001) compared with other senders.

**Table 3.** Comparing high-volume to all other senders: messaging behavior.

	Total sample (n=16,023)	High-volume message senders (n=1298)	All other message senders (n=14,725)	*P* value^a^
Messages per patient, mean (SD)	12.5 (20.4)	61.2 (43.6)	8.2 (7.5)	—^b^
Messages per patient, median (IQR)	6 (12)	49 (30)	6 (10)	—
Messaging months per patient, mean (SD)	3.9 (2.8)	9.5 (2.0)	3.4 (2.3)	<.001
Patients who had at least 1 high-intensity messaging episode, n (%)	2043 (12.8)	1160 (89.4)	883 (6)	<.001
Number of “high” messaging episodes per person, mean (SD)	0.3 (0.9)	2.4 (1.9)	0.1 (0.3)	<.001
Patients who sent at least 1 “long” message, n (%)	3755 (23.4)	872 (67.8)	2883 (19.6)	<.001
Number of “long” messages patients sent, mean (SD)	0.7 (2.4)	3.8 (13.0)	0.3 (1.0)	<.001
Total characters per person, mean (SD)	567.5 (375.3)	1020.9 (382.4)	527.5 (347.4)	<.001
Message-to-portal ratio, mean (SD)	0.18 (1.3)	0.52 (4.46)	0.15 (0.36)	<.001
Message-to-encounter ratio, mean (SD)	1.5 (2.7)	3.3 (6.5)	1.3 (1.9)	<.001

a Tests of statistical significance included *t* tests for continuous variables and chi-square tests for categorical variables.

b We did not conduct statistical tests comparing mean or median number of messages between high-volume and all other senders, as high-volume message senders were defined by disproportionately high messaging use and thus, by definition, sent a higher number of messages.

### Correlation and Convergence of Measures

All but 2 of the 5 measures were weakly correlated (Pearson correlation coefficients ranging from 0.08 to 0.33; Table 4), indicating that the 5 measures captured distinct aspects of messaging behavior. Secure messaging volume and high-intensity messaging episode measures were most highly correlated (Pearson correlation coefficient of 0.68).

**Table 4.** Correlation across measures.

Measures of high-intensity messaging use	Volume	Messaging episode	Length	Message-to-portal ratio	Message-to-encounter ratio
Volume^a^	1.00	0.68	0.11	0.14	0.31
Messaging episodes^b^	0.68	1.00	0.11	0.14	0.33
Length^c^	0.11	0.11	1.00	0.23	0.08
Message-to-portal ratio^d^	0.14	0.14	0.23	1.00	0.08
Message-to-encounter ratio^e^	0.31	0.33	0.08	0.08	1.00

a Volume was based on the aggregate number of messages sent by each patient during the observation period; high-volume senders were those who sent 33 or more messages during the observation period (threshold based on upper outlier calculated).

b Patients with at least 1 messaging episode (messages sent within 10 d of each other) with a number of messages per episode equaling or exceeding the upper outlier threshold calculated (≥8.5 messages per episode) were considered high-intensity.

c Patients with at least 1 message with number of characters equaling or exceeding the upper outlier threshold calculated (≥787.5 characters) were considered high-intensity message senders.

d Patients with ratios of 0.42 or greater (messages over number of patient portal logins) were considered high-intensity.

e Patients with ratios of 4.1 or greater (messages over number of encounters with the health care system) were considered high-intensity.

## Discussion

### Principal Findings

This observational study presented a profile of high-volume secure message senders across sociodemographic characteristics, health status, and messaging behaviors. We found that high-volume message senders comprised about 8% of message senders but accounted for nearly 40% of message volume. High-volume message senders were more often White and had a higher comorbidity burden. To more fully capture messaging behavior beyond aggregate volume, we developed 4 additional measures of messaging intensity. These revealed that high-volume message senders, compared with all other senders, were more likely to engage in sustained messaging, to send longer messages, to use the patient portal for functions beyond messaging, and to send more messages relative to their in-person health care encounters.

Our finding that all but 2 of the 5 measures of messaging intensity were weakly correlated underscores that patient-initiated messaging behaviors are nuanced. While a simple measure of aggregate messaging volume effectively captures patients who rely on messaging as a key form of communication with their clinicians, additional meaning can be derived from more granular measures of messaging behavior. Focusing on patients who meet multiple high-intensity messaging criteria could help resource-limited health systems identify patients with high communication needs who may benefit from additional clinical support. For example, patients who send long messages shortly after clinical encounters may benefit from a previsit agenda-setting tool to ensure that key concerns are addressed during the visit [49].

Our analysis found that high-volume senders sent, on average, 3 times as many long messages as all other message senders. Health systems and EMR vendors could also consider enhancing patient portal functionality to help clinicians more easily respond to lengthy messages. For example, clinicians may struggle to efficiently extract key details from long free-text messages [16,56]. A structured message template could help patients provide key information in an optimized format. Emerging AI-based approaches may further support message management by assisting with triage and prioritization of incoming messages based on urgency [57]. Some health systems also use AI to generate initial draft responses to patient messages, with drafts being reviewed and edited by clinicians before they are sent [58-60]. AI could be used to draft responses for only messages with a lower character count, protecting clinician time for responses to longer messages, which may be more complex and require greater clinical judgment.

Our finding that high-volume message senders had higher comorbidity burden (particularly with respect to cancer) and more extensive in-person health care use reinforces that high messaging volume may reflect greater health care needs. This highlights that efforts to manage secure messaging should focus not only on improving messaging efficiency but also on expanding health system capacity to meet the communication needs of patients with complex illnesses. A recent systematic review found that patients with chronic conditions find patient portal–based educational materials useful [61]. Health systems could embed curated educational materials within the patient portal to support patients with complex health conditions, providing information and referrals to community resources [62].

Current billing codes for clinician responses to patient messages are based solely on the amount of time spent replying to a message, without accounting for the clinical complexity or cognitive demands involved [22,23,32]. Our finding that a substantial proportion of messages come from patients with serious illnesses suggests that time-based billing codes may undervalue the clinical expertise and judgment required for high-quality messaging communication [23] when responding to messages from patients with complex care needs. Our findings may help inform refinements to billing models to more accurately reflect the level of effort and knowledge involved in supporting patients with complex care needs via secure messaging.

Importantly, input from clinicians, care teams, and patients is essential for developing effective strategies [63-65]. This should include understanding of which types of messages present the largest cognitive load or require the most effort to respond to, in which cases messaging is most helpful to patients, and identifying workflows and team structures that support optimal message handling. Costs of implementation and oversight requirements would need to be carefully considered with the implementation of any AI-based features. Ultimately, a multipronged approach to managing secure messaging volume, informed by patient needs, illness types, and communication preferences, can enhance both patient support and ease message-related burden on clinicians.

### Limitations

This study has several limitations. Our sample was constrained to patients receiving care from a single academic health system in one geographic region, limiting the generalizability of the findings. We did not capture health care that patients may have received elsewhere; patients may have sent messages to clinicians outside of our health system, and we may have underestimated their true messaging habits. We did not examine clinician factors that may influence patient messaging intensity, such as clinician response speed, response quality, or clinician encouragement to use secure messaging. Finally, as our analysis relied primarily on patient characteristics and information available within the EMR, we were unable to speak to external factors that influence messaging behaviors, such as digital literacy, social support, or preferences for other modes of communication. While our findings are limited to the inferences possible based on a single health system’s EMR, we present measures that can be readily replicated by other health systems to evaluate their patients’ unique messaging profiles and needs.

### Conclusions

Our study is the first to compare multiple approaches to defining high users of secure messaging within the patient portal using an empirical data-driven strategy (IQR method to identify outliers) that produces contextually based thresholds. We found that a small subset of patients accounted for a relatively large volume of messages (high-volume message senders) and exhibited distinct sociodemographic characteristics and messaging behavior regarding message length, when messages were sent, and messaging use in relation to patient portal use and health care encounters. Measures of messaging intensity were not highly correlated, suggesting that they reflect distinct dimensions of messaging behavior. Recognizing and responding to the different ways patients engage in secure messaging can help health systems enhance patient-clinician electronic communication and enable them to better face the challenges involved in managing patient message volume.

## Supplementary material

10.2196/93641 Multimedia Appendix 1 Mean, median, and upper threshold for each measure.

10.2196/93641 Multimedia Appendix 2 Description and rationale for high-intensity messaging measures.

## Abbreviations

ADI: Area Deprivation Index
aOR: adjusted odds ratio
CCI: Charlson Comorbidity Index
EMR: electronic medical record
*ICD-10*: *International Statistical Classification of Diseases, Tenth Revision*
