# Supplementary material for: The Role of Measurement in Identifying High-Intensity Secure Message Senders Among Older Adults: Observational Study
Source: J Med Internet Res. 2026 Oct 21;28:e93641. doi: 10.2196/93641 (PMC13686449; doi:10.2196/93641)
Supplement: Supplementary file 1 [file jmir-v28-e93641-s001.docx]

## **Multimedia Appendix 1. Mean, Median, and Upper Threshold for Each Measure**

| Measure | Mean (SD) | Median (IQR) | Upper Threshold ^a^ |
| --- | --- | --- | --- |
| Volume (number of messages) | 12.5 (20.4) | 6 (12) | 33 |
| Messaging episodes (messages in an episode ^b^) | 3.3 (6.1) | 2 (3) | 8.5 |
| Length (characters) ^c^ | 194 (281) | 272.8 (271.6) | 787.5 |
| Message-to-portal ratio | 0.18 (1.3) | 0.11 (0.14) | 0.42 |
| Message-to-encounter ratio | 1.5 (2.7) | 0.8 (1.5) | 4.1 |
| ^a^ The upper threshold was determined as: quartile 3 + 1.5 × IQR. Any values equaling or exceeding this upper threshold for each measure were considered high-intensity.  ^b^ For the messaging episodes measure, patients with at least one messaging episode with a number of messages per episode equaling or exceeding the upper threshold were considered high-intensity message senders.  ^c^ For the length measure, patients with at least one message with number of characters equaling or exceeding the upper threshold were considered high-intensity message senders. | | | |
