# Supplementary material for: The Role of Measurement in Identifying High-Intensity Secure Message Senders Among Older Adults: Observational Study
Source: J Med Internet Res. 2026 Oct 21;28:e93641. doi: 10.2196/93641 (PMC13686449; doi:10.2196/93641)
Supplement: Supplementary file 2 [file jmir-v28-e93641-s002.docx]

**Multimedia Appendix 2. Description and Rationale for High-Intensity Messaging Measures**

| Measure Name | Measure Description | Measure Rationale |
| --- | --- | --- |
| Volume ^a^ | Messaging volume is defined as the aggregate number of messages sent by each patient during the observation period. Patients sending an aggregate number of messages equal to or exceeding the upper threshold calculated using the interquartile rage method (IQR) method ^b^ are referred to as “high-volume” message senders. | Aggregate yearly volume is consistent with previous studies, ^c^ is simple to compute, and can be readily applied by health systems for identifying high-intensity message senders who may benefit from targeted interventions. |
| Messaging episodes | Messaging episodes are defined as concentrated periods of high-intensity messaging separated by breaks of 10 days or more. We selected a 10-day washout period to delineate episodes to conservatively allow five days for clinician response, since previous work had found that clinicians on average take three days to respond to a majority of their messages ^d^ and with an additional 2 days to account for non-business days. We allow an additional five days for subsequent patient response.  Within each episode, we count number of messages and identify high-intensity episodes using the IQR method; high-intensity episodes are messaging episodes with a disproportionately high number of messages. Patients with one or more high-intensity messaging episodes are categorized as “high-intensity” for this measure. | This measure is intended to identify patients who experience periods involving excessive messaging during a short time frame. The availability of such a measure could help health systems proactively identify patients initiating an intense messaging episode for targeted outreach and support during the episode. |
| Length | Length is determined by the number of characters per message. Patients with one or more messages with a disproportionately high number of characters using the IQR method are considered “high-intensity” for this measure. | We focus on message length as operationalized by character count as a proxy for messaging complexity, given that longer messages are likely more complicated or at a minimum, require more clinician time to read and process. |
| Message-to-portal ratio | The ratio of number of secure messages in relation to number of patient portal logins during the observation period. Patients whose ratio is identified as an outlier by the IQR methods are considered “high-intensity”. | This measure captures disproportionate messaging relative to portal use. By using outlier analysis, we identify patients who are primarily utilizing the patient portal to send messages (for communication or electronic care delivery navigation) as opposed to logging in to the patient portal for other reasons. |
| Message-to-encounter ratio | The ratio of the number of secure messages to the number of in-person encounters incurred during observation period. Patients whose ratio is identified as an outlier by the IQR methods are considered “high-intensity”. | This measure captures disproportionate messaging relative to the number of encounters with the healthcare system. We identify patients who are sending a substantially larger volume of messages compared to the number of encounters they have with the health system. |
| ^a^ Message volume is the primary measure of high-intensity message sending used to identify our main group of interest for analysis.  ^b^ The upper threshold was determined as: quartile 3 + 1.5 × IQR. Any values exceeding this upper bound were classified as upper outliers and thus “high-intensity”.  ^c^ Previous studies using similar methods: Cronin RM, Davis S, Shenson J, Chen Q, Rosenbloom S, Jackson G. Growth of secure messaging through a patient portal as a form of outpatient interaction across clinical specialties. Applied clinical informatics. 2015;6(02):288-30; Bryan M, Norton D, Birstler J, Chen G, Cruz L, Hanrahan L. Resource utilization among portal users who send messages: a retrospective cohort study. Wis Med J. 2020;119(1):26-32; Heisey-Grove DM, McClelland LE, Rathert C, Tartaglia A, Jackson K, DeShazo JP. Associations between patient health outcomes and secure message content exchanged between patients and clinicians: retrospective cohort study. Journal of Medical Internet Research. 2020;22(10):e19477.  ^d^ Source: Huang M, Fan J, Prigge J, Shah ND, Costello BA, Yao L. Characterizing patient-clinician communication in secure medical messages: retrospective study. Journal of medical Internet research. 2022;24(1):e17273. | | |
